# Mass spectrometry dataset on apo-SOD1 modifications induced by lipid aldehydes

**DOI:** 10.1016/j.dib.2020.105850

**Published:** 2020-06-12

**Authors:** Lucas S. Dantas, Alex Inague, Adriano Britto Chaves-Filho, Sayuri Miyamoto

**Affiliations:** Departamento de Bioquímica, Instituto de Química, Universidade de São Paulo, SP, Brazil

**Keywords:** PTM, Lipid aldehydes, Lipid electrophiles, Secoaldehydes, Oxysterol, SOD1

## Abstract

Metal-deficient Cu,Zn-superoxide dismutase (apo-SOD1) is associated with the formation of SOD1 aggregates that accumulate in ALS disease. The data supplied in this article support the accompanying publication showing SOD1 modification and aggregation induced by lipid aldehydes [1]. Here, we present the LC-MS/MS dataset on apo-SOD1 modification induced by seven different lipid aldehydes: 4-hydroxy-2-hexenal (HHE), 4-hydroxy-2-nonenal (HNE), 2-hexen-1-al (HEX), 2,4-nonadienal (NON), 2,4-decadienal (DEC) or secosterol aldehydes (SECO-A or SECO-B). Modified protein samples were digested with trypsin and sequenced by a LC coupled to a Q-TOF instrument. Protein sequencing and peptide modification analysis was performed by Mascot 2.6 (Matrix Science) and further validated by manual inspection. Mass spectrometry data (RAW files) obtained in this study have been deposited to MassIVE and the observed peptide-aldehyde adducts can be used in further studies exploring SOD1 modifications *in vivo*.

Specifications table

**Table:** 

Subject	Biochemistry
Specific subject area	Protein post-translational modifications, protein aggregation, lipid peroxidation, lipid electrophiles
Type of data	Figures
How data were acquired	Raw data were acquired on nanoAcquity UPLC system (Waters, United States) coupled to a Q-TOF mass spectrometer instrument (TripleTOF6600 Sciex, United States) using Analyst TF 1.7 for data-dependent acquisition.
Data format	Raw Analyzed
Parameters for data collection	Human recombinant apo-SOD1 was incubated with seven different lipid aldehydes to determine their effect on protein modification and aggregation.
Description of data collection	To understand how different lipid aldehydes modify apo-SOD1, we collected samples and submitted them to trypsin digestion and protein sequencing by LC-MS/MS.
Data source location	Institution: Department of Biochemistry, Institute of Chemistry, University of Sao Paulo City/Town/Region: Sao Paulo, SP Country: Brazil
Data accessibility	Processed data are available with the article and raw data are available on repository Repository name: MassIVE Data identification number: MSV000085309 Direct URL to data: https://doi.org/doi:10.25345/C5JH79 or https://massive.ucsd.edu/ProteoSAFe/dataset.jsp?accession=MSV000085309
Related research article	Dantas, L.S., Viviani, L.G., Inague, A., Piccirillo, E., Rezende, L., Ronsein, G.E., Augusto, O., Medeiros, M.H.G., Amaral, A.T., Miyamoto, S., Lipid aldehyde hydrophobicity affects apo-SOD1 modification and aggregation. Free Radic Biol Med XXX (accepted for publication)

## Value of the data

• The data show the characterization of apo-SOD1 lipoxidation sites induced by seven biologically relevant lipid aldehydes.
• These data can be useful for researchers studying protein lipoxidation.
• These data can be useful for studies investigating protein post-translational modifications induced by lipid peroxidation products.

## 1 Data description

**Fig. 1:**
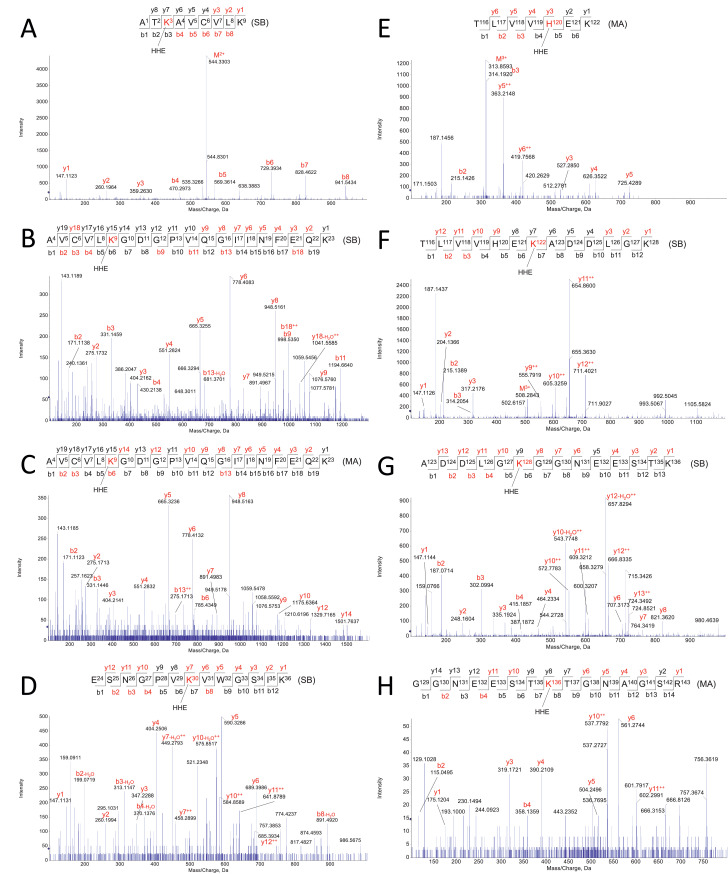
*Apo-SOD1 modifications induced by HHE.* Schiff-base (SB) and Michael adduct (MA) modifications were observed in the following residues: K3-SB (A), K9-SB (B), K9-MA (C), K30-SB (D), H120-MA (E), K122-SB (F), K128-SB (G), K136-SB (H).

**Fig. 2:**
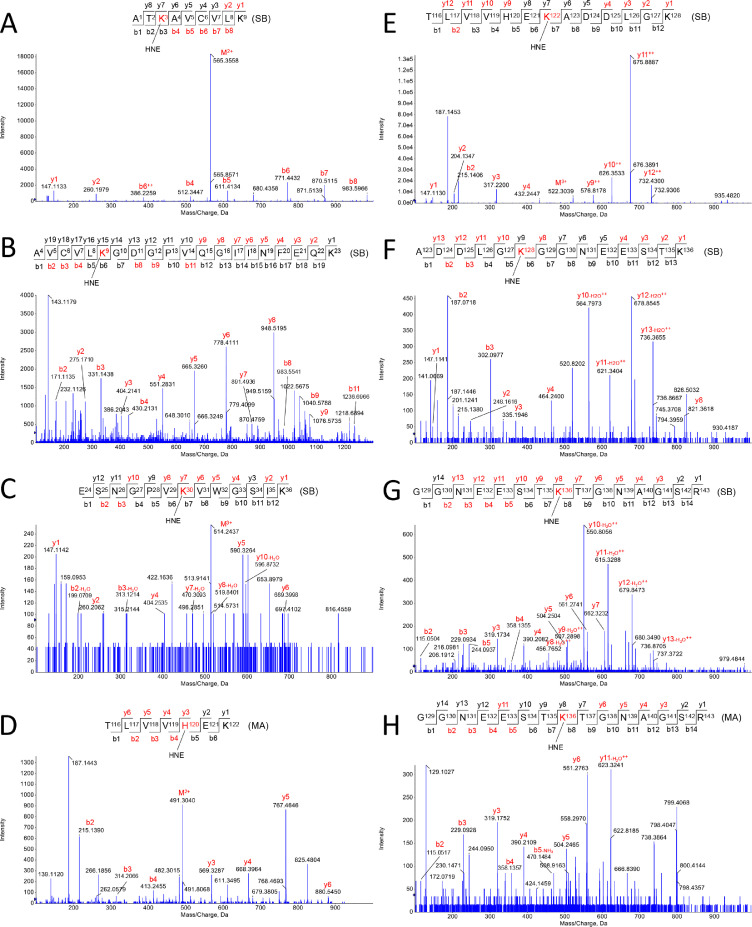
*Apo-SOD1 modifications induced by HNE.* Schiff-base (SB) and Michael adduct (MA) modifications were observed in the following residues: K3-SB (A), K9-SB (B), K30-SB (C), H120-MA (D), K122-SB (E), K128-SB (F), K136-SB (G), K136-MA (H).

**Fig. 3:**
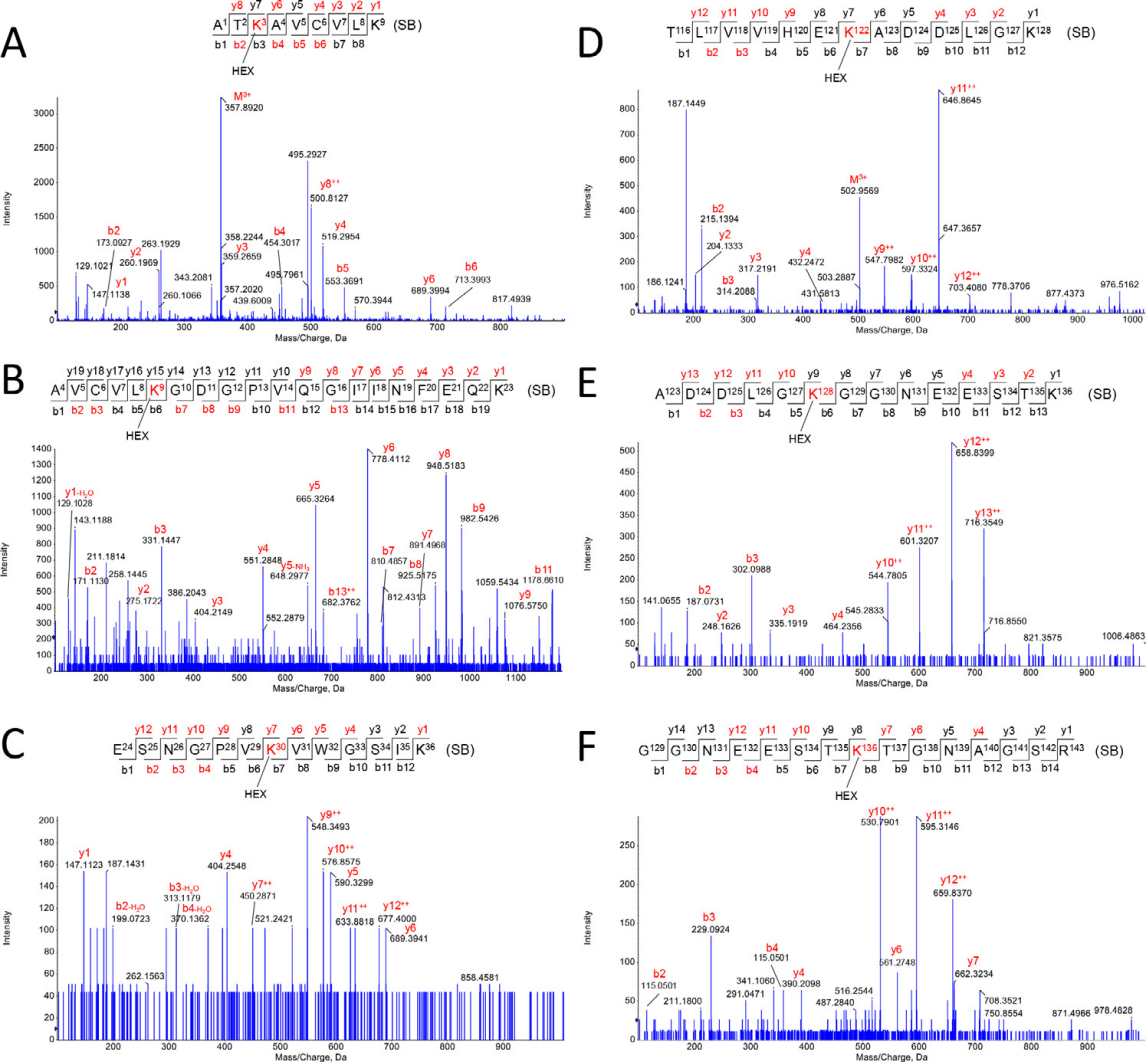
*Apo-SOD1 modifications induced by HEX.* Schiff-base (SB) and Michael adduct (MA) modifications were observed in the following residues: K3-SB (A), K9-SB (B), K30-SB (C), K122-SB (D), K128-SB (E), K136-SB (F).

**Fig. 4:**
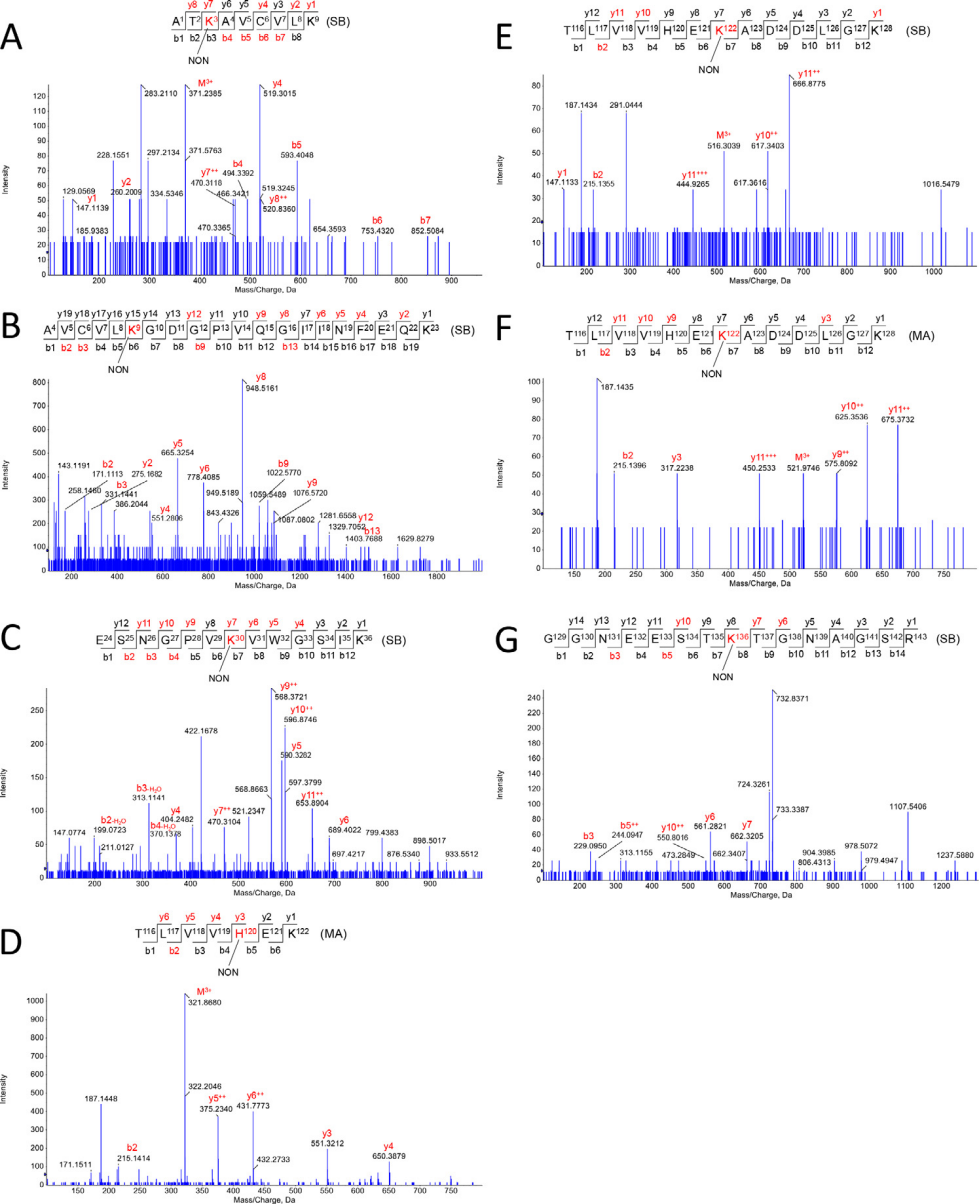
*Apo-SOD1 modifications induced by NON.* Schiff-base (SB) and Michael adduct (MA) modifications were observed in the following residues: K3-SB (A), K9-SB (B), K30-SB (C), H120-MA (D), K122-SB (E), K122-MA (F), K136-SB (G).

**Fig. 5:**
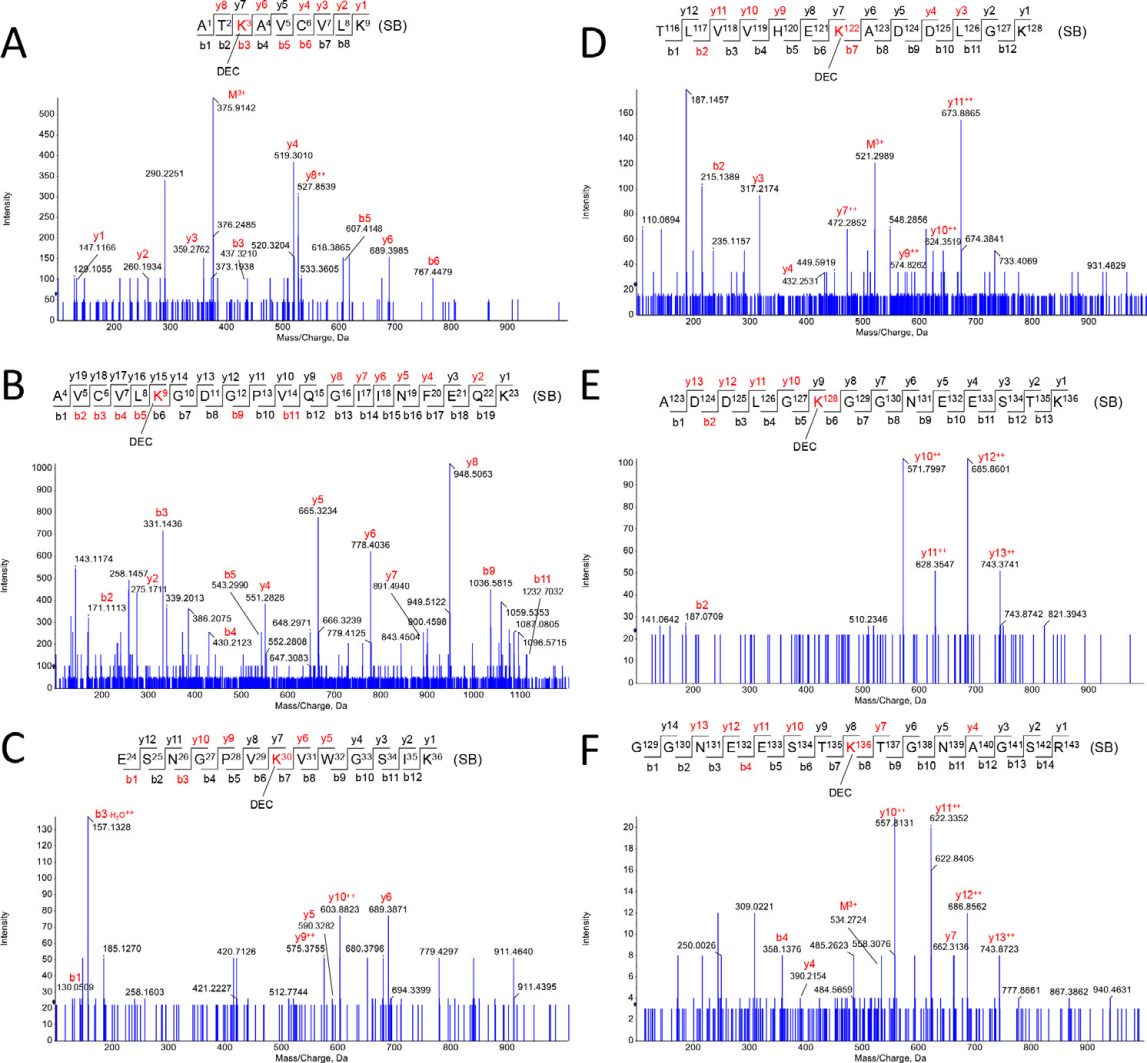
*Apo-SOD1 modifications induced by DEC.* Schiff-base (SB) and Michael adduct (MA) modifications were observed in the following residues: K3-SB (A), K9-SB (B), K30-SB (C), K122-SB (D), K128-SB (E), K136-SB (F).

**Fig. 6:**
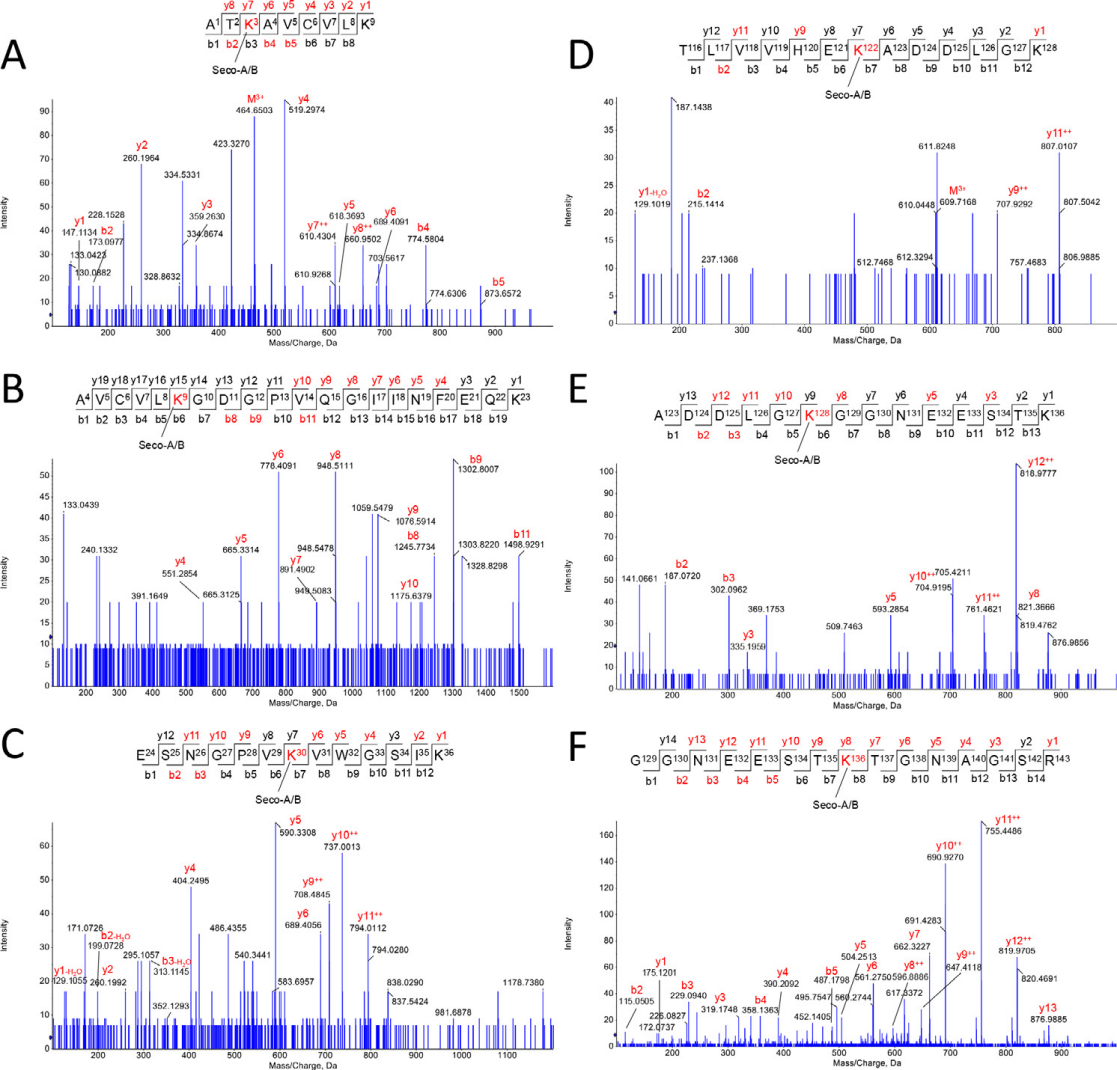
*Apo-SOD1 modifications induced by SECO-A or SECO-B.* Schiff-base (SB) and Michael adduct (MA) modifications were observed in the following residues: K3-SB (A), K9-SB (B), K30-SB (C), K122-SB (D), K128-SB (E), K136-SB (F).

This dataset contains raw and processed LC-MS/MS data on the characterization of apo-SOD1 lipoxidation sites induced by five different 2-alkenals (4-hydroxy-2-hexenal, HHE; 4-hydroxy-2-nonenal, HNE; 2-hexen-1-al, HEX; 2,4-nonadienal, NON; and 2,4-decadienal, DEC) and two cholesterol derived aldehydes (3β-hydroxy-5-oxo-5,6-secocholestan-6-al, SECO-A; and 3β-hydroxy-5β-hydroxy-B-norcholestane-6β-carboxaldehyde, SECO-B). Before proteomic analysis, a reduction step using sodium borohydride was applied to stabilize aldehyde-protein adducts. Modified proteins were then submitted to bottom-up proteomic analysis workflow using a high-resolution Q-TOF instrument (TripleTOF6600, Sciex) coupled to a nano-LC system. Protein lipoxidation sites were mapped using Mascot Server 2.6.1 and confirmed by manual inspection. Lipoxidation sites were searched considering the formation of Schiff base (SB) and Michael addition (MA) adducts with the side chains of Lys, His and Cys residues. SOD1 sequence coverage obtained by Mascot analysis was greater than 99%. Several modifications have been detected. Mass errors between theoretical and experimentally detected peptide adducts were below 10 ppm. Figs. 1–6 depict annotated MS/MS mass spectra showing the characterization of peptide adducts with HHE, HNE, HEX, NON, DEC and SECO-A or SECO-B, respectively.

## 2 Experimental design, materials and methods

### 2.1 Materials

4-hydroxy-2-hexenal (HHE) and 4-hydroxy-2-nonenal (HNE) were purchased from Cayman Chemical (Ann Arbor, MI, USA). *Trans*-2-hexen-1-al (HEX), *trans,trans*-2,4-nonadienal (NON), and *trans,trans*-2,4-decadienal (DEC) were purchased from Sigma (St. Louis, MO, USA). The 3β-hydroxy-5-oxo-5,6-secocholestan-6-al (SECO-A) and 3β-hydroxy-5β-hydroxy-B-norcholestane-6β-carboxaldehyde (SECO-B) were synthesized as previously described [2,3]. Aldehyde stock solutions (2.5 mM) were prepared in isopropanol. Human recombinant Cu,Zn-superoxide dismutase (SOD1) and its apo-form were prepared as described by Dantas et al. [4] Sequencing grade trypsin was obtained from Promega (Madison, WI, USA) and RapiGest SF Surfactant was acquired from Waters (Milford, MA, USA).

### 2.2 Incubation with lipid aldehydes

Aliquots of 20 μL of apo-SOD1 (final conc. 10 μM) were incubated with 20 μL of each aldehyde (final conc. 250 μM) and 160 μL of 50 mM phosphate buffer, pH 7.4, containing 150 mM NaCl and 100 μM diethylenetriamine pentaacetate (DTPA) at 37 °C for 24 h, with gentle agitation using Thermomixer (Eppendorf AG, Hamburg, Germany).

### 2.3 Protein digestion

After incubation, samples were first reduced with sodium borohydride (NaBH_4_, 5 mM), for 1 h at room temperature and then, submitted to disulfide reduction with dithiothreitol (DTT, 5 mM), for 30 min at 60 °C and Cys alkylation with iodoacetamide (15 mM), for 30 min at room temperature. Protein digestion was done with proteomic-grade trypsin (Promega, Madison, WI, USA) at a 1:100 (w/w) ratio, for 18 h at 37 °C, using RapiGest SF Surfactant (Waters, Milford, MA, USA).

### 2.4 LC-MS/MS analysis

Peptide mixture was analyzed by a LC-MS/MS system consisted of a nanoAcquity UPLC system (Waters Corp., Milford, MA, USA), coupled to a quadrupole-time-of-flight (Q-TOF) mass spectrometer (TripleTOF6600 Sciex, United States), as described previously [4]. First, samples were desalted on the trapping column (Waters, nanoAcquity Trap column, 180 µm × 20 mm; 5 µm) using 1% solvent B at a flow rate of 10 µL/min for 2 min under isocratic conditions. Peptides were then separated on a C18 analytical column (Waters nanoAcquity UPLC, 75 µm × 150 mm; 3.5 µm) using a gradient of 0.1% aqueous formic acid (mobile phase A) and 0.1% formic acid in acetonitrile (mobile phase B). Chromatographic separation was done at a flow rate of 400 nL min^−1^ for a total run time of 97 min according to a gradient shown below. Column temperature was kept at 35 °C. Sample injection volume was 2 µL.

**Table:** 

Time (min)	% A (0.1% formic acid in water)	% B (0.1% formic acid in acetonitrile)
0	99	1
60	65	35
61	10	90
73	10	90
74	99	1

Peptides were infused into the TripleTOF6600 instrument through a nano-ESI source (Sciex, Framingham, MA). The nano-ESI source was equipped with a nano-ESI emitter tip (New Objective). The mass spectrometer parameters were:

**Table:** 

Ion Source Parameters	Settings
Ion spray voltage floating (ISVF)	2400 V
Curtain Gas (CUR)	20
Interface heater (IHT)	120
Ion source gas 1 (GS1)	3
Ion source gas 2 (GS2)	0
Declustering potential (DP)	80 V

Tandem mass spectra were acquired by a data-dependent mode. TOFMS survey scan was set to the m/z range of 300–2000 and the accumulation time to 100 ms. Top 25 MS/MS spectra were acquired in the mass range of m/z 100–2000 with an accumulation time of 25 ms. The overall cycle time was 775 ms. Precursor ion selection criteria included charge state between + 2 and + 5 and ion intensity greater than 150 counts. Former fragmented precursor ions were excluded from reanalysis for 20 s. Fragmentation was performed using rolling collision energy with a collision energy spread of 5. For LC-MS/MS quality control we used 1 pmol/µl stock solution of beta-galactosidase, which was prepared according to manufacturer`s instruction (LC/MS peptide calibration kit P/N 4,465,867), pre-digested BSA or HeLa protein digest standard (Pierce, Thermo Scientific). Data acquisition was performed with Analyst TF 1.7 (Sciex). Mass spectrometry raw data have been deposited to the Mass Spectrometry Interactive Virtual Environment (MassIVE), with access via https://massive.ucsd.edu/ProteoSAFe/dataset.jsp?accession=MSV000085309.

### 2.5 Data analysis

Protein sequencing and modification analysis was performed with Mascot^Ⓡ^ software 2.6.1 version (Matrix Science Ltd., London, United Kingdom), using the following parameters:

**Table:** 

Mascot parameters	Settings
Database	SwissProt
Enzyme	Trypsin
Missed cleavages	Up to 4
Quantitation	None
Peptide tolerance	± 10 ppm
#^13^C	0
MS/MS tolerance	± 0.05 Da
Peptide Charge	2+, 3+, 4+
Monoisotopic	selected
Variable modifications	carbamidomethyl (C), oxidation (M) and aldehyde adducts
Data format	Mascot generic
Instrument	ESI-QUAD-TOF

In order to search for SOD1 lipoxidation sites, modifications corresponding to each aldehyde were added to the local Mascot server using the “Adding New Modification” button and filling with the following parameters:

**Table:** 

Adduct type	Δ Mass (Da)
Schiff base (SB) adducts with K or N-term A	
HHE	98.0731
HNE	140.1201
HEX	82.0782
NON	122.1095
DEC	136.1252
SECO-A or SECO-B	402.3497
	
Michael addition (MA) adducts with K, H, or C	
HHE	114.0680
HNE	156.1150
HEX	98.0731
NON	138.1044
DEC	152.1201
SECO-A or SECO-B	400.3341

Modified peptides identified by Mascot^Ⓡ^ were further validated by manual inspection. To identify the y and b fragments of the modified peptides and attribute their masses in MS/MS spectrum, we used the Bio Tool Kit microapp in PeakView^Ⓡ^ software. First, the protein sequence was digested *in silico* to create a list of theoretical peptides. Modified SOD1 peptide sequences found in MASCOT^Ⓡ^ software with their respective charges were selected in "Bio Tool Kit" and modifications corresponding to the mass of each aldehyde were added as variable modification. The software was settled to match the theoretical fragments to the ions in MS/MS spectrum with a match tolerance of 0.050 Da.

## CRediT authorship contribution statement

**Lucas S. Dantas:** Conceptualization, Investigation, Methodology, Formal analysis. **Alex Inague:** Methodology, Formal analysis. **Adriano Britto Chaves-Filho:** Methodology, Formal analysis. **Sayuri Miyamoto:** Conceptualization, Resources, Writing - review & editing, Supervision, Funding acquisition.

## Declaration of Competing Interest

The authors declare that they have no competing financial interests or personal relationships that could influence the work reported in this paper.
